# Acceptability of the ePOWER intervention: Managing previvors' cancer-related uncertainty and supporting decision making

**DOI:** 10.1016/j.pecinn.2025.100402

**Published:** 2025-05-10

**Authors:** Marleah Dean, Bethany Jowers, Claire Conley, Erica Camacho, Whitney Espinel, Kimberly A. Kaphingst

**Affiliations:** a Department of Communication, University of South Florida, Tampa, FL, USA; b Health Outcomes and Behavior Program, Moffitt Cancer Center, Tampa, FL, USA; c Department of Oncology, Georgetown University, Washington, DC, USA; d Department of Communication, University of Utah, Salt Lake City, UT, USA; e Huntsman Cancer Institute, University of Utah, Salt Lake City, UT, USA

**Keywords:** Uncertainty, Decision making, Multiple methods, Intervention, Genetic counseling

## Abstract

**Objective:**

Previvors—unaffected individuals who have increased risk of cancer due to a pathogenic or likely pathogenic variant in a gene—experience high levels of uncertainty, which is associated with negative outcomes. The ePOWER (empowering Preventive Options for Women Experiencing Risk) intervention is designed to help *BRCA1/2* previvors manage their cancer-related uncertainty and make informed health decisions. In this study, we assessed the acceptability of ePOWER using a multiple methods approach.

**Methods:**

Previvors (*N* = 24) completed individual, semi-structured interviews. Previvors first completed the Treatment Acceptability and Preference Scale (TAPS). Additionally, using a Learner Verification & Revision (LV&R) interviewing approach, we also elicited feedback on whether ePOWER was understandable, salient, and satisfactory to previvors. Acceptability was assessed by quantitative data (TAPS scores) and qualitative data (interviews). In analyzing the interview data and integrating the findings, deductive coding was utilized using LV&R categories and inductive thematic analysis was utilized to capture additional nuances from participants' evaluation.

**Results:**

Adequate acceptability was demonstrated by TAPS scores. 88 % of participants exceeded the a priori acceptability threshold (TAPS ≥3). Deductive coding using LV&R categories also confirmed ePOWER was visually appealing, understandable, persuasive, cultural appropriate, and fostered self-efficacy. Inductive thematic analysis expanded on the LV&R categories and identified two additional themes: (1) relatability and emotional support and (2) useful resource.

**Conclusion:**

ePOWER is an acceptable intervention to help previvors manage cancer-related uncertainty and support decision making.

**Innovation:**

The ePOWER intervention can be shared during healthcare appointments and then utilized continuously by previvors to manage uncertainty and facilitate decisions.

## Highlights

• Previvors are unaffected individuals who have increased risk of cancer due to a pathogenic variant in a gene.
• The ePOWER intervention helps previvors manage uncertainty and make decisions.
• The ePOWER intervention is an acceptable intervention.

## 1 Introduction

Previvors are unaffected individuals who have increased risk of cancer due to a pathogenic or likely pathogenic variant (P/LP variant) in a gene related to cancer development [1]. For example, previvors with a P/LP in the *BRCA1* gene have up to a 72 % lifetime breast cancer risk and up to a 44 % lifetime ovarian cancer risk; however, previvors with a P/LP in the *BRCA2* gene have up to a 69 % lifetime breast cancer risk and a 17 % lifetime ovarian cancer risk [2]. Previvors face unique psychosocial challenges and have distinct information needs [[3], [4], [5]]. First, previvors may experience intense uncertainty and fear about developing hereditary cancer in the future [6]. Second, previvors must navigate cancer risk management options (e.g., risk-reducing surgery versus increased surveillance) and family planning decisions (e.g., loss of childbearing and breastfeeding) that differ from the general population [[6], [7], [8]]. Furthermore, previvors have up to a 50 % risk to pass on their pathogenic variant to each future offspring [7,[9], [10], [11], [12], [13]], which may impact family planning [14]. In short, testing positive for a P/LP variant in *BRCA1/2* leads previvors to cope with chronic uncertainty [15].

### 1.1 Uncertainty and decision making

Uncertainty exists when information is probabilistic, ambiguous, or complex, resulting in being unsure of a possible future event [1]. Uncertainty is inherent to living with a *BRCA1/2* P/LP [3]. After a positive genetic test result, previvors often experience stress, anxiety, and uncertainty as they navigate multiple cancer risk management decisions over time [6,16]. Research has found previvors experience significant uncertainty when deciding between increased surveillance and preventive surgery [6,15], and when making family planning decisions [17,18]. Moreover, uncertainty can cause patients to feel less in control of their future and decrease their ability to identify coping resources [19,20]. In other words, previvors often struggle to make decisions about prevention and treatment because of uncertain information or limited evidence [15,[21], [22], [23]]. Previvors who are unable to manage this chronic uncertainty are at risk for heightened psychological distress over time [6,24], impacting their ability to make informed health decisions and lowering their quality of life [16,22,23].

### 1.2 Uncertainty management

Uncertainty health theorizing provides a robust theoretical framework for previvors' health information seeking and uncertainty management behaviors [25]. Uncertainty management involves making specific choices based on the perceived threat (i.e., future cancer diagnosis) and available information (e.g., genetic test results, information given by a clinician, or information found on social media) [26]. Previvors who perceive and appraise their uncertainty as a threat experience negative emotions and poor health outcomes [6,27,28]. However, these appraisals often change over time [6], requiring continued reassessment of cancer risk management decisions. Thus, interventions that address previvors' uncertainty over time by providing continually relevant health information and psychosocial support are direly needed [5,29].

Recent systematic and scoping reviews indicate a need for educational materials and interventions to support previvors [30,31], particularly addressing uncertainty [32,33]. Previous research has found information helps previvors increase knowledge and sense of control, manage uncertainty, and make cancer risk management decisions [1,4]. At the same time, uncertainty is a barrier for seeking information. Survey data revealed previvors (1) are unclear how to begin seeking information after testing positive, (2) struggle to find information across their healthcare journey, and (3) interface with conflicting information [3]. Thus, we developed an intervention to provide educational and psychosocial support for previvors navigating chronic, cancer-related uncertainty and cancer risk management decisions.

### 1.3 The ePOWER intervention

Our research team developed “ePOWER – empowering Preventive Options for Women Experiencing Risk.” This intervention was designed to increase female *BRCA1/2* previvors' knowledge of cancer risk management options, help them manage their chronic uncertainty, and assist them in making cancer risk management decisions. In our previous work, we found that previvors want a centralized resource for information [3], and previvors want information in the format of stories [4]. Thus, ePOWER is in booklet format (see, Appendix A, for example page) and includes health information, medically accurate narratives from previvors, empowering messages, and list-based next steps and resources (i.e., questions to ask clinicians, websites to visit, and support services and tools). Guided by our prior research [4,15], ePOWER utilizes uncertainty theorizing (i.e., Uncertainty Management Theory, Uncertainty in Illness Theory, Theory of Motivated Information Management) [25,26,[34], [35], [36]] and narrative persuasion [[37], [38], [39], [40]] to help previvors manage their chronic, cancer-related uncertainty and assist them in making cancer risk management decisions. More information on the development of ePOWER can be found published elsewhere [41].

Following best practices for developing a behavioral medicine intervention [42], the purpose of this study was to assess the acceptability of ePOWER. Specifically, we asked the following research question: *How do previvors evaluate the ePOWER intervention?*

## 2 Methods

### 2.1 Eligibility and recruitment

This study was approved by the University Institutional Review Board (#IRB005069). Eligible participants were 18-years or older, assigned female at birth, had not been diagnosed with cancer, and had tested positive for a pathogenic or likely pathogenic *BRCA1/2* variant. Participants were recruited through a cancer genetics clinic at an academic medical center. A certified genetic counselor queried the Cancer Institute's database to identify eligible participants and sent them an IRB-approved recruitment email. Interested participants completed the survey which confirmed eligibility, gathered demographic information, and listed possible scheduling options for interviews.

### 2.2 Data collection

Acceptability is the perception that an intervention is agreeable and satisfactory by the intended audience [43]. We assessed the acceptability of ePOWER in two ways. First, participants completed brief Qualtrics survey consisting of the Treatment Acceptability and Preference Scale (TAPS, see Appendix B) [44]. TAPS scores of 3 or higher indicated adequate intervention acceptability. Second, semi-structured interviews were conducted with previvors using a Learner Verification & Revision (LV&R) framework [45]. The main goal of LV&R is to elicit feedback on whether educational materials are understandable, salient, and satisfactory to the intended audience. If problems are identified, the LV&R procedure also indicates potential solutions.

Video interviews were conducted via Zoom, audio recorded, and professionally transcribed. The interview assessed if participants liked the ePOWER booklet and if they believed it addressed their psychosocial needs and supported their cancer risk management decision making (see Appendix C). Participants were compensated with a $30 gift card. Pseudonyms are used to throughout the manuscript. Twenty-four previvors participated in this study (see Table 1).

**Table 1:** Participants' demographics (*N* = 24).

Variable	Category	Number of participants
Age (Years)	18–24	1
	25–34	11
	35–44	8
	45–54	2
	55–64	1
	65+	1
Race/Ethnicity (Select all that apply)	White	22
	Hispanic, Latino, or Spanish	3
	Middle Eastern/North African	2
	Asian	3
Ashkenazi Jewish Heritage	Yes	3
	No	21
Sexuality	Heterosexual	20
	Bisexual	3
	Other*	1 *Participant self-described as demisexual
Relationship Status	Not in a relationship	8
	In a relationship, living apart	3
	In a relationship, living together	13
Highest Education Level Attained	Vocational	1
	Some college	2
	Associate's/bachelor's	14
	Graduate degree	7
Employment Type	Unemployed/homemaker/self-employed	4
	Student	2
	Employed part-time	5
	Employed full-time	13
Income	$100,000+	11
	$75,000–$99,999	2
	$50,000–$74,999	5
	$25,000–$49,999	3
	<$15,000	1
	Prefer not to answer	2
# of People in Household	1–2	10
	3–4	9
	5+	3
	Prefer not to answer	2
# of Children	0	9
	1–2	9
	3–4	5
	5	1
Insurance	Purchased directly	3
	Via employer	18
	Medicaid/Governmental	2
	TRICARE/Military	1
Time Since Genetic Testing (Years)	1–3	7
	3–5	1
	5–10	14
	10+	2
Cancer Risk Management (Select all that apply)	Self-breast exams	15
	Clinical breast exams	15
	Ovarian screening	9
	Skin screening	7
	Bilateral mastectomy	7
	Bilateral oophorectomy	6
# of Family Members Aware of their *BRCA1/2* Statuses	1–5	18
	5–10	5
	10+	1
# of Cancer Diagnoses in Family	0	9
	1	13
	2	2

### 2.3 Data analysis

Recruitment and interviews continued until theoretical saturation was reached—meaning no new insights, themes, or information emerged [46]. The first three authors analyzed the interview data in two ways. First, given the LV&R framework the research team utilized a direct content analysis approach [47]. Specifically, guided by the first author, the second and fourth authors organized participants' responses using the LV&R categories of attraction, comprehension, cultural acceptability, self-efficacy, and persuasion. The second and fourth authors tabulated “yes” or “no” responses for each of the categories and summarized key points, particularly regarding areas for improvement. The study team members then discussed the tabulations. The most frequent participant responses ultimately guided revisions. In other words, when several participants brought up the same idea, the study team discussed that suggestion and considered making that revision.

Yet, we realized deductive coding using LV&R categories did not fully capture participants' evaluation. As such, we also employed thematic analysis to analyze the transcripts [48], identifying additional themes that expanded [49] on the LV&R categories, which were not evident in the deductive coding. This process involved multiple steps. First, independently reading the interview transcripts again, the first three authors immersed themselves in the data to understand how previvors evaluated the intervention. We met several times throughout immersion to compare notes and initial reactions. Next, we coded six of the transcripts individually and generated initial codes by labeling and organizing the text. We then met to resolve any differences and consolidate codes. After this, we developed a codebook which the second and fourth authors utilized to code the remaining transcripts (*N* = 18), refining the codebook throughout. The first author reviewed the other authors' coding, and all three authors continually met to discuss coding. Once coding was finished, the first author assembled the codes into potential themes. The themes served as units of analysis and were detected based on Owen's thematic criteria of recurrence, repetition, and forcefulness [50]. Last, the first author kept a reflexive journal throughout the analysis process [51].

## 3 Results

**Fig. 1:**
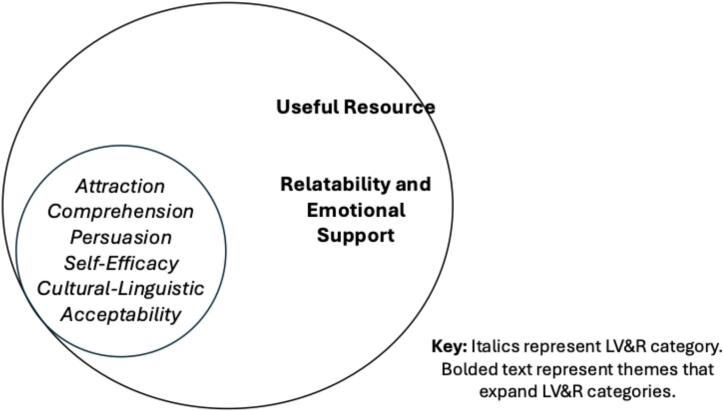
Integrated model of Learner Verification & Revision (LV&R) categories and inductive themes.

Following a multiple methods approach [52], we first report the acceptability scores from the TAPS, and second, explain how previvors evaluated the intervention using the LV&R codes and the identified themes that expand [49] on the LV&R categories (see Fig. 1).

### 3.1 Surveys: TAPS results

The average TAPS score was 3.55 (out of 4), indicating high acceptability. 88 % of participants exceeded our a priori acceptability threshold (≥3) [44].

### 3.2 Interviews: deductive coding using LV&R categories

Guided by LV&R categories, deductive coding demonstrated the intervention was attractive and comprehensive, would help previvors make cancer risk management decisions and manage their worry about hereditary cancer risk, and was culturally and linguistically appropriate.

#### 3.2.1 Attraction

Most participants (80 %) found the booklet attractive. These participants described the booklet as inviting and friendly, and several participants expressed they would pick up the booklet given the colors and overall graphic design. For example, Caitlin said, “I think they're calming colors. It looks like something that's made to be eye-catching yet comforting.” Alice agreed saying, “I think it's really a friendly kind of vibe.” Jillian shared, “I felt like if I had been reading it before getting any of my surgeries or knowing much about it, I feel like it would be kind of a positive feeling for me like, ‘I can do this.’”

Some participants remarked that the intervention was well-structured. For instance, participants felt the booklet covered key topics related to a previvor's healthcare journey (e.g., genetic testing and cancer risk management) in an organized format. For example, Caitlin stated, “I like how you do the next steps on every page as well.” Others appreciated how the sections were color-coded and had subtitles. At the same time, others struggled with the organization. Sybil said,

It wasn't clear to me the main message of each page, because there's a lot on each page…I do love the information, and I do love the stories. I think it was maybe just a little bit more about how it's organized in a focal point to the page.

Last, some participants suggested improving the booklet's attraction by changing the font from cursive to one easier to read and commented the booklet was text heavy, particularly the stories. Ayah said, “…It was just overwhelming, there's just so many words everywhere, it's hard to follow one thing.” Similarly, Alice shared the booklet was wordy but also noted that was helpful for information seekers. She explained,

I think some of the pages are really wordy, but I don't think that's necessarily a bad thing, especially when people are seeking information…I would want more information. I mean, that's just also the type of person I am, so this wouldn't bother me, per se, but maybe your layperson that's not in medicine, and this is a little bit really above their head and scary, maybe that's a little too much for them, but I do like the patient experiences and their thoughts and words.

#### 3.2.2 Comprehension

The LV&R category of comprehension assesses if the materials are understandable. Participants were asked to identify specific words or phrases that were difficult to understand. Participants stated the booklet was easy to understand with little jargon. Nicole said, “I didn't feel like there was any jargon that I didn't understand.” While most participants stated there was little jargon in the booklet, a few participants noted they were unfamiliar with the term “chemoprevention” and “previvor.” These participants suggested adding a glossary to the end of the booklet as a quick reference point. Also, some shared they were unfamiliar with some of the online resources (e.g., Stanford BRCA Tool) and social support groups (e.g., FORCE). For instance, Alice said, “I didn't even know about that Stanford tool that you could use…So I went on there, and I messed around with that today.” Likewise, Mackenzie shared, “I hadn't thought about support groups.” Finally, participants suggested revising the title to better represent the booklet's content.

#### 3.2.3 Persuasion

ePOWER was designed to help previvors manage their uncertainty and support decision making rather than persuading them to engage in a specific health behavior. Thus, participants were asked if they felt the booklet would help previvors make cancer risk management decisions. Several participants noted receiving the booklet along with their initial genetic testing results would be ideal. They shared ePOWER would help cope with their positive genetic test results as well as make cancer risk management decisions. For example, Jillian said, “I think that [ePOWER] would've been helpful…That's kind of my takeaway from this is I really like hearing real life stories and what people have done. That helped me make my decision.” Norah shared a similar perspective stating, “I think the information from it [ePOWER] will bring me to resources that will help me with making those decisions.” Carolina summarized, “I want a decision aid to help me understand my options and walk me through the process, and … I love that they're questions to help you think.”

Last, a few participants emphasized it would be helpful to return to ePOWER at different points during their healthcare journey (e.g., when making decisions about preventive surgery or family planning). A couple participants suggested revising ePOWER to serve as a checklist for making cancer risk management decisions.

#### 3.2.4 Self-efficacy

Participants who shared they had anxiety about a future cancer diagnosis were asked how the information would help them in managing their worry about their lifetime hereditary cancer risk. Kyra shared, “Every screening day is pretty anxious. I always think is today the day my life changes? Is it going to be today that it's the change of my life…I need the information to be the powerful one. So, I think having this would be helpful.” Additionally, others explained the personal stories and social support resources would help manage worry. Only one participant stated the information in the booklet would not help her because she experienced severe anxiety due to her mother's cancer diagnosis.

#### 3.2.5 Cultural-linguistic acceptability

When asked if the intervention was culturally and linguistically appropriate, all participants stated ePOWER's text and visuals did not make them feel uncomfortable. Additionally, participants liked the diversity of the characters and stated the booklet was inclusive in terms of body type and most racial/ethnic groups. For instance, Nadia said, “I like that it's inclusive of all different types of women.” Natasha expounded further saying,

I think that you did a really good job at different body shapes and different colors and different ethnicities. I think that you did a really good job at playing everyone into it because not everyone's skinny and not everyone has the boobs, and not everyone is tiny.

Carolina summarized, “I think it's very approachable. I like – I don't know how you call it – the characters [graphic design animations] as opposed to actual people… Although, I will say it's kind of trendy in the design world right now, so that might change over time.” Two participants noted representation was needed for Asian, Muslim, and Jewish ethnic groups. Another participant suggested adding someone with gray hair.

### 3.3 Interviews: expanding LV&R categories through thematic analysis

Inductive thematic analysis also identified two themes that expanded [49] on the LV&R categories, which were not evident in the deductive coding.

#### 3.3.1 Relatability and emotional support

We found the ePOWER intervention, particularly the stories, resonated with participants' lived experiences. For example, Caitlin said, “I look at it, and I say, “Oh, this applies to me because I could easily put myself in any one of their shoes… It almost mirrors exactly what I had gone through.” When discussing one of the narratives about cancer risk management decision making, Jillian commented, “What are you going to do now? … I love that because that's exactly how I felt. What do I do?” Furthermore, when reviewing the page about talking to one's doctor, Nadia said, “I think this did list exactly what I kind of talked about with my doctor.”

In addition, participants also noted they connected with the stories in ePOWER, which provided emotional support. For instance, some participants shared the booklet made them feel not alone. Sybil explained, “I think the number one thing it would've done is just give me some comfort. I'm not alone. There's a whole community of people that are dealing with this, thinking about this.” Similarly, Ayah expressed,

I feel [when I look at ePOWER], number one, I'm not alone…That's one thing I saw when I first opened it. I was like, “Okay, this is a space for me. This is talking directly to me.” …That's the first impression that I got was I'm not alone—a reminder that there are plenty of people out there who are going through the same thing.

Furthermore, Margot stated, “I like reading through the stories and saying, ‘Okay, it's more common than I think outside of my family. There are more people than just me, and my family going through this, or we are there.’”

#### 3.3.2 Useful resource

We learned that the ePOWER intervention was a helpful resource for previvors—going beyond helping facilitate decisions—and could be utilized to assist previvors in preparing for clinical appointments. For example, Margot said, “I think this booklet is a really good combination of all the things you need to know regarding if you have this certain gene.” Norah shared, “I learned a lot in this pamphlet. I thought I knew a good chunk.” For example, participants stated the booklet would help them prepare for clinical appointments. For example, Emiko shared she also liked the Q&A section saying, “I like the end, too, because it actually made you think of questions you should ask your provider and be thinking of when you actually went in.” Indeed, Natasha stated she planned to use some of the questions in her upcoming appointment. She said,

I do plan on filling that [Q&A section] out. I go back to my high-risk doctor on Tuesday, and I actually wanted to have it filled out before I went to her to see if it would help me talk to her better on figuring out the next steps because I feel like I'm in a limbo stage of not actually knowing what we're doing.

One participant suggested having the resources on the resource page be hyperlinked or include a QR to make it easier to access them.

### 3.4 Integration of quantitative and qualitative data

In an attempt to further integrate our results, we identified participants reporting low acceptability (TAPS score < 3, *N* = 3) and re-examined those participants' transcripts, paying particular attention to their coded LV&R categories and inductive themes. However, we found no systemic difference between those with low v. adequate TAPS scores. Thus, it is possible that the factors that drive low acceptability are idiosyncratic.

## 4 Discussion and conclusion

This multiple methods study [52] demonstrates the ePOWER intervention is acceptable and elucidates the ways in which it may assist previvors through the provision of information and narratives. Guided by uncertainty theorizing [25,26] and narrative persuasion [37,38] frameworks, the ePOWER intervention addresses a current gap in *BRCA1/2* decision support resources [53,54]. Results demonstrate key takeaways that can guide future work.

### 4.1 Discussion

Quantitative data from the TAPS questionnaire indicated adequate acceptability, and inductive data provided further insights about most and least acceptable aspects of ePOWER including guidance for revisions. The LV&R approach [55] provided specific elements to consider, identified areas for modification, and ensured the intervention is suitable, culturally appropriate, and actionable [55]. Specifically, participants stated ePOWER was comprehensive, which provided *BRCA1/2*-related information and would have helped facilitated their decisions. As such, this intervention fills a previously identified gap that while information-gathering is an effective uncertainty management strategy [1], younger previvors need more information related to genetic testing, cancer prevention, and support [56].

Furthermore, ePOWER addresses previous research's conclusions that previvors have distinct medical and psychosocial information needs across different stages of their healthcare journey [4]. In fact, in a survey of 101 previvors, we found uncertainty was a barrier to information needs; previvors expressed not knowing how to start seeking information, did not understand medical jargon, and struggled to disentangle conflicting information between healthcare providers and the Internet [3]. In the current study, participants shared ePOWER would assist them in making decisions and coping with genetic testing results, an encouraging finding.

Thematic analysis further clarified exactly how previvors would use ePOWER to manage their cancer-related uncertainty through narrative relatability and emotional support. This finding would have not been identified without the use of both data analysis approaches [52]. In this study, participants connected with the stories, which provided emotional support from those in similar situations, and participants who shared they had anxiety or worry about being diagnosed with cancer in the future explained the stories and social support resources would help manage worry and process their emotions. This is promising as extant research has found previvors turn to their social support networks such as family members and friends to cope with their inherited cancer risks [57,58] as well as clinicians for supportive communication to manage their cancer-related uncertainty [1]. In other words, the stories may serve as a substitute for emotional support when it is not available in a previvor's existing network. Moreover, because ePOWER utilizes actual language from previvors in the stories, this may have increased participants' identification with the stories, enhanced their ability to process emotions, and assisted them in enacting the behaviors modeled in the intervention [38,40,41,59].

### 4.2 Innovation

The ePOWER intervention is innovative in two ways. First, rooted in uncertainty health theorizing which emphasizes chronic uncertainty management, it assists previvors in managing their hereditary cancer risks *over time* [6,25,60]. As noted earlier, most decision support tools provide support only during the pre- or post-genetic counseling sessions [53]. This is not sufficient, particularly for women who choose increased surveillance and thus manage heightened uncertainty surrounding screening appointments. Second, the ePOWER intervention can provide previvors with information and decision-making support *outside* of clinical care. Genetic counselors can share the booklet during appointments such as when returning genetic test results, but previvors can also refer to the information and stories at home when needed. This can help manage uncertainty and facilitate decisions when prompted during their healthcare journey (e.g., when waiting for diagnostic test results from a breast MRI, after learning about a family member's ovarian cancer diagnosis, or when deciding if and when to have children).

### 4.3 Conclusion

One limitation of this study is the sample. Despite maximum variation sampling to select our participants, most participants were White, non-Hispanic, college educated and had health insurance through a current or former employer or union. We are continuing to explore and utilize strategies for recruiting more diverse patients in the future [61,62]. Additionally, participants who volunteered to participate in this study may be more aware of their information needs and uncertainty management struggles. Thus, next steps include incorporating participants' feedback and testing this intervention's outcomes during genetic counseling sessions upon the return of genetic test results [42].

In sum, this study assessed the acceptability of the ePOWER intervention and found that it is acceptable to previvors. Importantly, it is innovative because it seeks to support previvors outside of the clinical encounter over time, addressing a significant gap in psychosocial and informational support. Feedback from the interviews is currently guiding modifications to the ePOWER intervention (see Table 2). Our long-term goal is to help previvors manage chronic, cancer-related uncertainty and make informed cancer risk management decisions. Thus, next steps include a randomized trial testing the effectiveness of the intervention in increasing previvors' knowledge about cancer risk management options, managing their cancer-related uncertainty, supporting their cancer risk management decision-making, and enhancing their overall psychological well-being and quality of life.

**Table 2:** Summary of previvors' suggested changes to ePOWER intervention.

LV&R categories		
Category	Suggested change	Modifications being made
Attraction	• Changing title font from cursive to one easier to read • Reduce amount of text, particularly with the stories	• Edited font title • Did not reduce text in stories; did not want to lose rich, narratological elements of the stories
Comprehension	• Make the main message on each page clearer • Add a glossary at the end as a quick reference point • Revising the title to better represent the whole booklet	• Revised headings for each stage of a previvor's healthcare journey • Plan to add a glossary with medical terms (e.g., chemoprevention) • Revised title to “ePOWER – empowering Prevention Options for Women Experiencing Risk: A practical guide for managing risk and making decisions”
Persuasion	• Make ePOWER like a checklist for making cancer risk management decisions	• Did not change; not the intended goal of the booklet though it doesn't keep previvors from using it in this way
Self-Efficacy	• No suggestions provided	• NA
Cultural-Linguistic Acceptability	• Add Asian, Muslim, and Jewish ethnic groups • Add someone with gray hair	• Did not change; future iterations of the booklet will diverse ethnic groups further


Inductive themes		
Theme	Suggested change	Modifications made
Relatability and Emotional Support	• No suggestions provided	• NA
Useful Resource	• Utilize ePOWER (particularly Q/A section at the end) to prepare for clinical appointments • Add hyperlinks in PDF or a QR code for social support resources	• No changes made; doesn't keep previvors from utilizing it in this way • Plan to hyperlink resources or include a QR code

## CRediT authorship contribution statement

**Marleah Dean:** Writing – review & editing, Writing – original draft, Visualization, Validation, Supervision, Resources, Project administration, Methodology, Investigation, Formal analysis, Data curation, Conceptualization. **Bethany Jowers:** Writing – review & editing, Methodology, Investigation, Formal analysis. **Claire Conley:** Writing – review & editing, Validation, Investigation. **Erica Camacho:** Writing – review & editing, Methodology, Investigation, Formal analysis. **Whitney Espinel:** Writing – review & editing, Methodology, Investigation. **Kimberly A. Kaphingst:** Writing – review & editing, Validation, Methodology, Investigation.

## Ethics statement

All procedures were approved by the University of South Florida IRB (STUDY005069) and the University of Utah IRB (IRB_00163979).

## Funding

NA

## Declaration of competing interest

Dr. Conley has received grant funding from 10.13039/100004319Pfizer. The rest of the authors declare that they have no known competing financial interests or personal relationships that could have appeared to influence the work reported in this paper.

## Appendix A Supplementary data

Supplementary material 1 Image 1

Supplementary material 2 Image 2

Supplementary material 3 Image 3
