# Supplementary material for: Acceptability of the ePOWER intervention: Managing previvors' cancer-related uncertainty and supporting decision making
Source: PEC Innov. 2025 May 10;6:100402. doi: 10.1016/j.pecinn.2025.100402 (PMC12143645; doi:10.1016/j.pecinn.2025.100402)
Supplement: Supplementary file 1 — Supplementary material 1 [file mmc1.pdf]

After Genetic Testing

After testing positive for a BRCA mutation, you may want to come up with a plan to identify cancer at an earlier, more treatable stage, or even prevent a future cancer diagnosis. Talk to your doctor to create a plan. It is okay if your plan changes over time. You can also talk with other previvors to learn what decisions they made and to receive emotional support. To help you create a plan with your doctor and receive support from women, read these stories.

Co-Creating an Action Plan With Your Doctor

"When the genetic counselor told me I tested positive for BRCA, I was shell-shocked, and I said to her, "Well, what do I do now?" She told me two things. "Go see an oncologist and go to the FORCE website." Online I learned about my options for surveillance, preventive surgery, and chemoprevention. I trusted my doctor and the information she gave me because she was knowledgeable about BRCA. Having a doctor that gives you information is key, but having a doctor who also listens to what you want is just as important. My doctor explained steps I could take to either identify cancer at an early, treatable stage, or to prevent cancer, and knowing these options reduced my anxiety. Then we wrote a plan together and even scheduled a couple follow-up appointments so she could check in with me.

Yes, I have a higher risk for developing cancer during my lifetime, but I am empowered because I created an action plan with my doctor to take care of myself."

Next Steps:

- Think about how you would like your doctor to be involved in making decisions about your health:

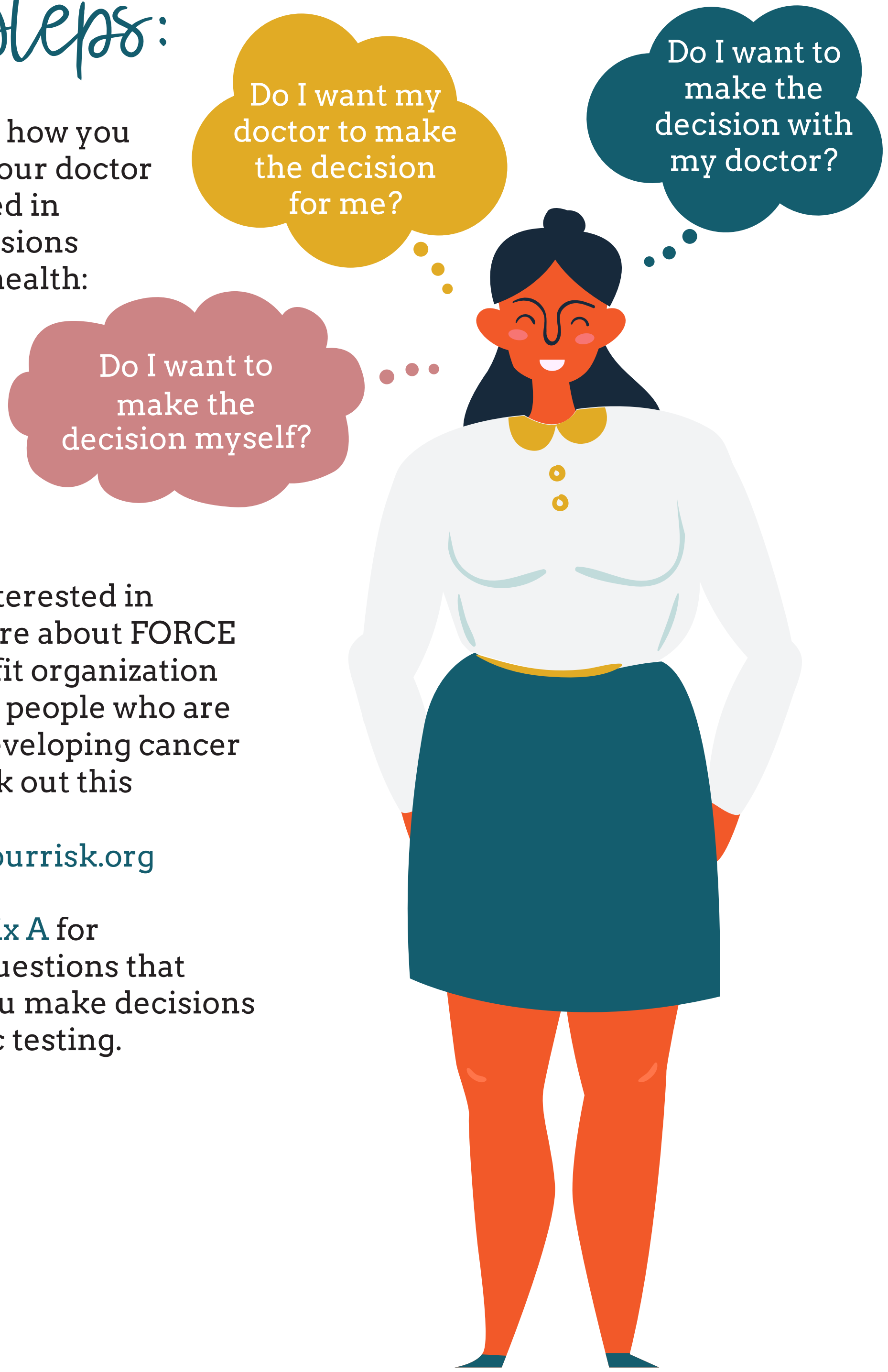

- If you are interested in learning more about FORCE—a non-profit organization dedicated to people who are at risk for developing cancer—then check out this website: www.facingourrisk.org

- See Appendix A for additional questions that may help you make decisions after genetic testing.
