# Supplementary material for: Acceptability of the ePOWER intervention: Managing previvors' cancer-related uncertainty and supporting decision making
Source: PEC Innov. 2025 May 10;6:100402. doi: 10.1016/j.pecinn.2025.100402 (PMC12143645; doi:10.1016/j.pecinn.2025.100402)
Supplement: Supplementary file 2 — Supplementary material 2 [file mmc2.docx]

***Adapted Treatment Acceptability and Preference Scale (TAPS) for Female BRCA1/2 Previvors about ePOWER Intervention***

1. How **effective** do you think the ePOWER booklet will be for learning about your cancer risk management options?
2. How **effective** do you think the ePOWER booklet will be for deciding how to manage your cancer risks (e.g., preventive surgery vs. surveillance)?
3. How **effective** do you think the ePOWER booklet will be for managing your worry/fear about being diagnosed with cancer in the future?
4. How **logical** does the ePOWER booklet seem to you?
5. How **suitable** does the ePOWER booklet seem to you?
6. How **willing** are you to use to the ePOWER booklet?
7. How **willing** are you to share to share the ePOWER booklet with family members?

Reference

Martorella, G., Gélinas, C., & Purden, M. (2014). Acceptability of a web-based and tailored intervention for the self-management of pain after cardiac surgery: the perception of women and men. *JMIR Research Protocols*, *3*(4), e3175.
