# Supplementary material for: Acceptability of the ePOWER intervention: Managing previvors' cancer-related uncertainty and supporting decision making
Source: PEC Innov. 2025 May 10;6:100402. doi: 10.1016/j.pecinn.2025.100402 (PMC12143645; doi:10.1016/j.pecinn.2025.100402)
Supplement: Supplementary file 3 — Supplementary material 3 [file mmc3.docx]

***Individual, Semi-Structured Interview Guide with Female BRCA1/2 Previvors about ePOWER Intervention***

Attraction

1. Looking at the cover, would you want to pick up this booklet and read it?
   1. Why or why not?
2. Looking over the booklet, how much does it hold your attention?
3. What did you like about the booklet?
   1. What did you dislike about the booklet?
   2. What changes do you suggest to improve the booklet?

Comprehension

1. Tell me, in your own words, what is this booklet all about?
   1. What are the key sections which are most important to you?
2. What changes would you suggest to make the information in the booklet clearer?
   1. Were you any points of confusion? Any jargon?
3. How accurate does the graphic novel seem to you?
   1. What changes would you suggest to make the graphic novel more accurate?
4. How true to life does it seem?
   1. Did the information resonate with you and your life experiences?

Self-Efficacy

1. How confident are you that you can manage their worry about your lifetime risk of hereditary cancer?
   1. Do you worry about a future cancer diagnosis, and if so, would this booklet help manage that worry/anxiety?
   2. Did the booklet create reminders of the importance of a *BRCA1/2* diagnosis / feeling of urgency/severity of having a *BRCA1/2* diagnosis?

Cultural-Linguistic Acceptability

1. How do you feel when you look at the booklet?
   1. What emotions do you experience/feel when looking at the booklet?
   2. Is there anything in the material that makes you uncomfortable? What is that?
2. Are there any pictures that are not acceptable for you or your family? What are those?
3. Are there any words or phrases that are not acceptable for you or your family? What are those? What changes would you suggest?

Persuasion

1. How helpful would the booklet be in helping you made decisions about your health care?

Improvement

1. How might we improve the booklet to better suit your needs in making health decisions (or health journey stage)?
   1. Is there any information missing?
2. What would make the booklet more useful?

*Dissemination*

1. Would you share this booklet with a female biological relative? Why or why not?
   1. What changes to the booklet would make you more likely to share the information with a relative?
   2. What would be your hope in sharing it with them?
2. How could we share this booklet to help others?
   1. When do you think it would have been most helpful to get this booklet?

**CLOSING THE INTERVIEW**

Those are all of my questions, but before we end, *is there anything else you would like to share with me about the booklet?* Perhaps something we did not discuss that you think is important to this conversation?

Thank you for your time in participating in this interview! We greatly appreciate your feedback and believe your opinions will assist in improving the booklet.

Reference

Chavarria, E. A., Christy, S. M., Simmons, V. N., Vadaparampil, S. T., Gwede, C. K., & Meade, C. D. (2021). Learner verification: A methodology to create suitable education materials. *HLRP: Health Literacy Research and Practice*, *5*(1), e49-e59.
